# Ezetimibe is effective when added to maximally tolerated lipid lowering therapy in patients with HIV

**DOI:** 10.1186/1476-511X-6-15

**Published:** 2007-06-13

**Authors:** Matthew T Bennett, Kevin W Johns, Gregory P Bondy

**Affiliations:** 1 Department of Medicine, University of British Columbia, Vancouver, Canada; 2 Immunodeficiency Clinic-HIV Metabolic Clinic, St. Paul's Hospital, Vancouver, Canada

## Abstract

To determine the efficacy and safety of adding ezetimibe to maximally tolerated lipid lowering therapy in patients with HIV dyslipidemia.

Retrospective analysis of lipid parameters was conducted for 33 patients with HIV who had been prescribed ezetimibe 10 mg per day.

Mean total cholesterol was reduced 21% (p < 0.001). Mean LDL was reduced 35% (p < 0.001). Mean HDL increased 8% (p = 0.038). Mean triglyceride was reduced 34% (p = 0.006). Mean Apolipoprotein B100 was reduced 33% (p = 0.043). No adverse events occurred.

Ezetimibe appears safe and effective in patients with HIV when added to maximally tolerated doses of lipid lowering therapy.

## Background

Patients with HIV have an increased risk of coronary artery disease [1]. Part of this risk may be due to the hyperlipidemia associated with anti-retrovirals [1,2]. Often the lipid goals of patients in this group are not achieved by the therapy recommended in the current lipid lowering guidelines [3].

Ezetimibe which blocks the intestinal absorption of dietary cholesterol and bile acid absorption[4], has been effective in optimizing lipid levels when added to traditional therapy in non-HIV positive patients [5,6]. In HIV positive patients two studies have assessed the efficacy of ezetimibe. Coll et al. showed that ezetimibe monotherapy decreases LDL as effectively as fluvastatin monotherapy in HIV positive patients [7]. Negredo et al. showed that LDL was also reduced when ezetimibe was added to pravastatin monotherapy[8].

It is not known if adding ezetimibe to maximally tolerated lipid lowering therapy, whether it be a fibrate, a highly potent statin or both, in HIV positive patients will further optimize the serum lipid profile. Furthermore, the safety profile of these combinations is not known in this population.

33 HIV positive patients in our center were started on ezetimibe, as they were not at target despite maximally tolerated lipid lowering therapy. Serum lipid concentrations before and after adding ezetimibe were analyzed to assess its efficacy.

## Methods

We analysed the effect on the lipid profile of the addition of ezetimibe in patients with HIV.

All patients were seen at the Immunodeficiency Clinic (IDC)-HIV Metabolic Clinic at St. Paul's Hospital, Vancouver, BC, Canada. This is a tertiary referral center where over 700 patients receive care for HIV associated metabolic disorders. Patients who were treated with Ezetimibe during the time period January 2003 to May 2006 were included. Ezetimibe was initiated only after they were on the maximum tolerated doses of standard lipid lowering therapy.

The effect of ezetimibe on the serum concentrations of total cholesterol, LDL, HDL, triglycerides and apolipoprotein B were analysed. In addition, adverse events as defined by an elevation in AST or ALT over 5 times ULN or CK over 10 times normal or any symptom requiring discontinuation were documented.

Ethics approval was granted by the Ethics Review Board at St. Paul's Hospital.

### Patients

40 patients seen in the IDC-HIV Metabolic Clinic had been prescribed ezetimibe 10 mg per day orally. Seven of these patients were not included (2 had alterations in their other medications, 3 took ezetimibe for less than 4 weeks (discontinued due to reasons other than adverse events such as the inability to pay for the medication), and 2 had insufficient data).

Of the remaining 33 patients, 8 had type 2 diabetes mellitus, 16 had hypertension, 9 had prior vascular disease, 10 previously smoked and 3 of the patients were current smokers. 31 of the patients were male and the average age at the start of ezetimibe therapy was 51.4 years (range 39–76).

24 of the patients were on HMG-CoA reductase inhibitors (2 on pravastatin, 15 on rosuvastatin and 7 on atorvastatin), 17 of the patients were on fibrates (all fenofibrate), 2 of the patients were on Niacin and 4 of the patients were on salmon oil.

24 of the patients were taking protease inhibitors, 25 were taking NNRTIs, 29 were taking NRTIs, 1 was taking T20 and 1 subject was on no anti-retroviral therapy.

The serum triglycerides were significantly elevated in 8 of the patients precluding the calculation of serum LDL concentration. Only 7 of the patients had apolipoprotein B measurements both before and after the institution of ezetimibe.

Univariate analysis was used to compare the differences between the pre and post treatment using the Wilcoxon rank sum test for paired non-parametric samples. SPSS version 14.0 was used to analyse the data.

## Results

Mean total cholesterol was reduced from 6.95 mmol/L to 5.51 mmol/L (21% reduction, p < 0.001). Mean LDL was reduced from 4.05 mmol/L to 2.63 mmol/L (35% reduction, p < 0.001). Mean HDL increased from 1.07 mmol/L to 1.16 mmol/L (8% increase, p = 0.038). Mean triglyceride was reduced from 6.22 mmol/L to 3.85 mmol/L (34% reduction, p = 0.006). Mean apolipoprotein B100 was reduced from 1.45 g/L to 1.09 g/L (33% reduction p = 0.043). The average duration of ezetimibe therapy was 79.6 days (range 33–193 days). No adverse events occurred.

**Figure 1:**
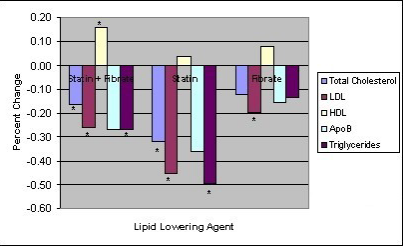
Ezetimibe add-on therapy: Effect according to baseline lipid agent * p < 0.05.

The patients were analyzed according to their baseline lipid lowering therapy. The results are described in figure 1. When ezetimibe was added to maximally tolerated doses of a statin and a fibrate, reductions were seen in serum concentrations of total cholesterol (16%, p = 0.004), LDL (26%, p = 0.008), and triglycerides (28%, p = 0.021). Furthermore the serum HDL concentration increased in this group (17%, p = 0.036). When ezetimibe was added to maximally tolerated doses of statin monotherapy, reductions were also seen in serum concentrations of total cholesterol (32%, p = 0.001), LDL (45%, p = 0.008) and triglycerides (49%, p = 0.033) in the subgroup of patients on maximally tolerated doses of statin monotherapy. When ezetimibe was added to maximally tolerated doses of fibrate monotherapy, the serum LDL concentration decreased by 20% (p = 0.043). In each of these subgroups, the concentrations of total cholesterol, LDL, HDL, apolipoprotein B and triglycerides improved. Due to small sample size only the results mentioned above were statistically significant.

## Conclusion

Following our analysis we conclude that adding ezetimibe improves the lipid profile in patients with HIV on maximally tolerated doses of lipid lowering therapy.

In our group of 33 patients we found significant reductions in serum concentrations of total cholesterol, triglycerides, LDL and apolipoprotein B100 following the addition of ezetimibe. Serum concentrations of HDL also rose significantly following this intervention. These gains were achieved without any adverse events.

Our findings are concordant with studies which have assessed the efficacy of ezetimibe in non-HIV populations [5,6]. As one of the proposed mechanisms of dyslipidemia in patients with HIV is due to ARV therapy[9], we felt it was necessary to demonstrate the efficacy of ezetimibe in this group.

Only two studies have described the use of ezetimibe in patients with HIV. Coll et al compared ezetimibe monotherapy to fluvastatin monotherapy in 20 HIV positive patients. Serum concentrations of LDL were reduced by 20% with ezetimibe monotherapy [7]. As our practice is to add ezetimibe to other lipid lowering therapy, only 3 patients in our study were on ezetimibe monotherapy. The serum LDL concentration was reduced by 32% in this subgroup. This result was not statistically significant owing to the small number of patients in this subgroup.

Negredo et al. added ezetimibe in 19 patients with HIV who were not at target while on pravastatin. After 24 weeks of combination therapy of ezetimibe and pravastatin, 61.5% of patients achieved an LDL less than 3.36 mmol/L (130 mg/dl). At baseline there were 14 patients in our study on statin therapy who had a serum LDL concentration greater than 3.36 mmol/L. 9 of these patients (64%) achieved an LDL of less than 3.36 mmol/L following the addition of ezetimibe.

**Figure 2:**
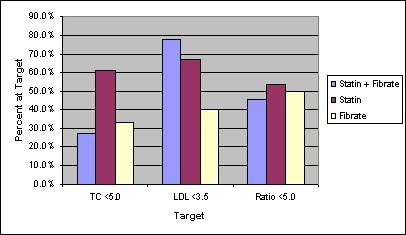
Ezetimibe add-on therapy: Percent of patients reaching targets for moderate risk according to baseline lipid agent.

In our center, in the absence of consensus guidelines for the treatment of dyslipidemia in patients with HIV, we currently treat HIV positive patients to at least moderate risk lipid profile targets (total cholesterol concentration of less than or equal to 5.0 mmol/L, an LDL concentration of less than or equal to 3.5 mmol/L and serum total cholesterol to HDL ratio of less than or equal to 5.0). Prior to ezetimibe therapy, none of the patients met all targets despite maximally tolerated lipid lowering therapy (none of the patients met the total cholesterol target, 6 of 25 patients met the LDL target and 4 of 33 patients met the total cholesterol to HDL ratio target). Following the addition of ezetimibe the total cholesterol was reduced to less than or equal to 5.0 mmol/L 13 of 33 patients (39%). The LDL concentration was reduced to less than or equal to 3.5 mmol/L in 15 of 25 patients (60%). The ratio of total cholesterol to HDL ratio was reduced to less than or equal to 5.0 in 15 of 33 patients (45%). The results were then analyzed according to baseline lipid lowering therapy. Figure 2 describes which patients met moderate risk lipid targets following the addition of ezetimibe despite not meeting them at baseline.

The current published guidelines recommend diet and exercise counseling, considering altering the ARV regimen or adding lipid lowering medications for dyslipidemia in patients on ARV therapy [3]. A statin is suggested for LDL elevations and a fibrate is suggested for serum triglycerides elevations. The current guidelines do not yet recommend the use of ezetimibe therapy in this group.

Our study is the first to analyze the efficacy of adding ezetimibe to maximally tolerated doses of the lipid lowering therapy including highly potent statins, fibrates and combinations of a statin and a fibrate. Significant improvements in the lipid profile following the addition of ezetimibe were seen. Furthermore, there were no adverse events. We conclude that if the lipid targets are not met after maximally tolerated doses of lipid lowering therapy with a statin and/or fibrate, ezetimibe is safe and effective.

## Competing interests

The author(s) declare that they have no competing interests.

## Authors' contributions

MB was the principle author of the paper, participated in design of the project and aided in data acquisition. KJ was the primary collector of data by means of chart review, conducted statistical analysis and edited the manuscript. GB designed the project, aided in data acquisition and was the principal editor of the manuscript. All authors read and approved the final manuscript.
